# Lateral septal nucleus, dorsal part, and dentate gyrus are necessary for spatial and object recognition memory, respectively, in mice

**DOI:** 10.3389/fnbeh.2023.1139737

**Published:** 2023-03-31

**Authors:** Ying-Ke Jiang, Fei-Yuan Dong, Yi-Bei Dong, Xin-Yi Zhu, Lu-Hui Pan, Lin-Bo Hu, Le Xu, Xiao-Fan Xu, Li-Min Xu, Xiao-Qin Zhang

**Affiliations:** ^1^Department of Pharmacology, Health Science Center, Ningbo University, Ningbo, Zhejiang, China; ^2^Ningbo Women and Children’s Hospital, Ningbo, Zhejiang, China

**Keywords:** lateral septal nucleus (LSD), dorsal part, dentate gyrus, recognition memory, c-Fos, lesion

## Abstract

**Introduction:**

Cognitive impairment includes the abnormality of learning, memory and judgment, resulting in severe learning and memory impairment and social activity impairment, which greatly affects the life quality of individuals. However, the specific mechanisms underlying cognitive impairment in different behavioral paradigms remain to be elucidated.

**Methods:**

The study utilized two behavioral paradigms, novel location recognition (NLR) and novel object recognition (NOR), to investigate the brain regions involved in cognitive function. These tests comprised two phases: mice were presented with two identical objects for familiarization during the training phase, and a novel (experiment) or familiar (control) object/location was presented during testing. Immunostaining quantification of c-Fos, an immediate early gene used as a neuronal activity marker, was performed in eight different brain regions after the NLR or NOR test.

**Results:**

The number of c-Fos-positive cells was significantly higher in the dorsal part of the lateral septal nucleus (LSD) in the NLR and dentate gyrus (DG) in the NOR experiment group than in the control group. We further bilaterally lesioned these regions using excitotoxic ibotenic acid and replenished the damaged areas using an antisense oligonucleotide (ASO) strategy.

**Discussion:**

These data reinforced the importance of LSD and DG in regulating spatial and object recognition memory, respectively. Thus, the study provides insight into the roles of these brain regions and suggests potential intervention targets for impaired spatial and object recognition memory.

## 1. Introduction

Cognitive function is the process of recognizing and acquiring knowledge. Furthermore, it involves a series of psychological and social behaviors, such as learning, memory, and emotion. Cognitive impairment refers to the abnormality of advanced brain processing associated with learning, memory, and judgment, which triggers severe impairment of learning and memory and obstacles to social activity, accompanied by related pathological changes (Dolan, 2002). This series of physical and psychological changes greatly affect the life quality of individuals, which has added heavy burdens to families and social economy. However, the specific mechanisms responsible for cognitive impairment in different behavioral paradigms remain to be elucidated. Brain neural circuits and networks form the basis of advanced cognitive functions. Specific brain regions and different types of neurons are involved in neural circuit formation (Fernandez et al., 2018). Therefore, understanding the neural activity in specific brain regions of different behavioral paradigms is important for providing potential targets to regulate cognitive function.

Previous studies show that there are many brain regions associated with the regulation of object and spatial cognition, especially the hippocampus and the perirhinal cortex (PRh) (Jarrard, 1995; Opitz, 2014; Cinalli et al., 2020). The PRh is crucial for object recognition, the ability to distinguish between the novel and familiar stimulus (Outram et al., 2022). Anatomy of the brain have shown the PRh and hippocampus to be strongly connected in the rodents (Condé et al., 1995). The hippocampus receives main cortical input from the lateral and medial entorhinal cortex. Object information from the PRh to the hippocampus is transmitted primarily *via* the lateral perforating path, with fibers terminating in the dentate gyrus (DG) and subfields cornu ammonis (CA)3 and CA1. Moreover, spatial information is transmitted *via* the medial entorhinal cortex. Thus, the DG, CA1, and CA3 are of vital importance of object and spatial cognition. The dorsal part of the lateral septal nucleus (LSD) and cingulate cortex are found the projections received from the hippocampus (Rajasethupathy et al., 2015; Wirtshafter and Wilson, 2021). The piriform nucleus (Pir), adjacent to PRh, is thought to be the main cortical region encoding odor identity, which is important for odor perception and spatial memory (Poo et al., 2022). Therefore, we choose CA1, CA3, and DG of the hippocampus, the LSD, the cingulate cortex (Cg)1 and Cg2 of the anterior cingulate gyrus (ACC), Pir and PRh.

Behavioral tests are widely used in numerous aspects of neuroscience, particularly in animal model assessments and physiological mechanisms related to cognitive impairment. Various behavioral paradigms have been developed to study the cognitive function of rodents, including maze tests, such as the Morris water maze, Barnes maze, Y maze, and Eight-arm maze. Others include open field tests, such as novel location recognition (NLR), novel object recognition (NOR), and fear conditioning test. Among them, NLR and NOR are the most common behavioral paradigms for studying spatial and object recognition in rodents, based on their instinct to explore novel things (Antunes and Biala, 2012). Importantly, these two behavioral paradigms have the characteristics of simple experimental equipment, short experimental time, no external reward or punishment as a motivation, and no need for food deprivation, enabling animals to perform cognitive behaviors in a state of free activity and more closely simulate human learning and memory behavior (Grayson et al., 2015). Additionally, the NOR and NLR tests, an evaluation method in the long-term study of brain injury diseases, could be repeated in the same batch of mice. The mice could continue to prefer to explore novel objects and locations (Li et al., 2014). Thus, we assessed spatial and object recognition by NLR and NOR in mice and identified the shared or distinct brain regions underlying different recognition memories.

In the present study, the NLR or NOR test comprised two phases: training (familiarization) and testing. In the training session, the mice were presented with two identically shaped and similarly sized objects for familiarization; a novel (experiment) and familiar (control) object/location were then presented during testing. Mice were subjected to NLR or NOR and sacrificed for immunostaining quantification of c-Fos in eight different brain regions. The number of c-Fos-positive cells was significantly higher in the LSD, NLR, and DG in the NOR group than in the control group. To investigate the specific role of the LSD and DG in object and spatial recognition, we used an excitotoxic ibotenic acid lesion strategy to damage these two regions bilaterally in mice. An antisense oligonucleotide (ASO) strategy that converts astrocytes to functional neurons by depleting the ribonucleic acid (RNA)-binding polypyrimidine tract binding protein (PTBP1) was used to replenish the damaged areas (Qian et al., 2020). We identified the principal brain regions activated in spatial and object recognition memory by studying the correlations between behavioral changes and specific brain regions before and after lesions.

## 2. Materials and methods

### 2.1. Animals

Male Institute of Cancer Research (ICR) mice (age: 8 weeks) were obtained from the Shanghai Sino-British SIPPR/BL Lab. A total of 102 male ICR mice were used, with 54 used for NOR and 48 for NLR. Mice were housed under standard conditions at 22°C and a 12 h light: dark cycle with free access to food and water.

All experiments were conducted following the National Institutes of Health Guide for the Care and Use of Laboratory Animals and approved by the Animal Care and Use Committees of Ningbo University, China (Reference Number: 10099). All efforts were made to minimize the mice’s suffering and reduce the number of mice used for the experiments.

### 2.2. Drugs

The drugs used in the present study were ibotenic acid (Med Chem Express, Shanghai, China), ASO, and nonsense oligonucleotide (NSO) (Beijing Genomics Institute, Guangdong, China). Ibotenic acid is a powerful neurotoxin that produces excessive Ca^2+^ by activating glutamate receptors, resulting in neuronal cell death (Dunnett et al., 1991). Ibotenic acid was dissolved in 0.9% saline to a concentration of 6 μg/μl. Recently some studies have achieved successful neuronal conversion of glial cells through the repression of PTBP1, which encodes a key RNA-binding protein. ASO strategy is able to implement this function by downregulating PTBP1. The adult-born functional neurons integrate into endogenous circuits and improved behavior of mice. Compared with other approaches, ASO strategy could overcome the potential risks posed by permanent downregulation of PTBP1 as it can be paused (Maimon et al., 2021; Contardo et al., 2022). The ASO sequence for mouse PTB was 5′-GGGTGAAGATCCTGTTCAATA-3′. The sequence of NSO was 5′-TGTCGGAGTCGTGAA-3′ and was used as a control. ASO and NSO were dissolved in ddH_2_O to a concentration of 4 μg/μl.

### 2.3. Stereotaxic injection of drugs

The mice were anesthetized with 0.5% pentobarbital sodium (80 mg/kg) and positioned in a stereotaxic apparatus (68030, RWD, China). Body temperature was maintained at 37°C using a heating pad. Drugs (ibotenic acid or ASO) were injected using a glass micropipette with a tip diameter of 15–20 μm through a small skull opening (<0.5 mm^2^). We injected 0.3 μl/side of ibotenic acid into the LSD (+0.2 mm AP, ±0.5 mm ML, −2.6 mm DV) or DG (−2.16 mm AP, ±1.0 mm ML, −1.86 mm DV) at a rate of 0.05 μl/min. After injection, the needle was left for 5 min before being carefully lifted. For the control group, 0.9% saline was injected into the same brain region. Mice were allowed to recover for 7 days before undergoing behavioral tests, which were performed over 2 days. After that, we injected 0.5 μl/side of ASO or NSO into the LSD or DG about 9 days after the first injection. Furthermore, behavioral tests were performed 15 and 30 days after the last injection.

### 2.4. Behavioral tests

**FIGURE 1:**
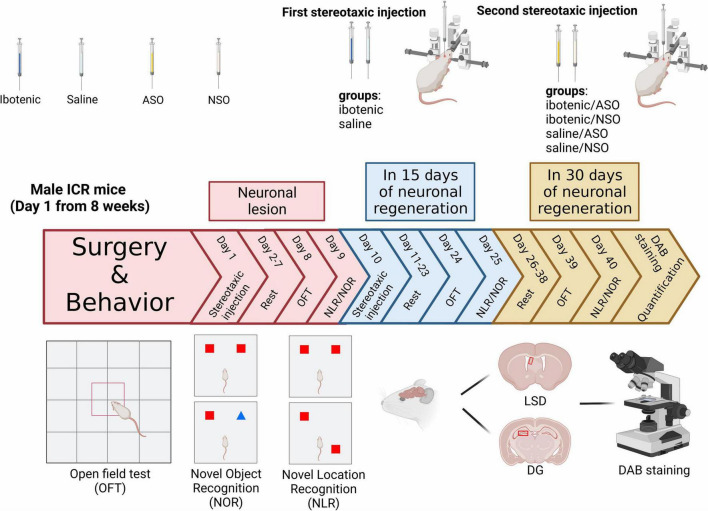
Timeline of the study and experimental design. ASO, antisense oligonucleotide; NSO, nonsense oligonucleotide; OFT, open field test; NOR, novel object recognition; NLR, novel location recognition; LSD, the dorsal part of the lateral septal nucleus; DG, dentate gyrus; DAB staining, 3,3′-diaminobenzidine staining. Created with https://biorender.com.

Behavioral tests were conducted in the order shown in Figure 1, with mice being given a minimum of 7 days to recover from the surgery. The open field test (OFT) was used to assess the locomotor activity of the mice post-surgery. The NLR and NOR were used to test spatial and object recognition memory, respectively. All tests were performed and analyzed by experimenters blinded to the animal group and drug treatments.

#### 2.4.1. Open field test

The OFT was used to evaluate locomotor activity (Kraeuter et al., 2019). The OFT was conducted in a black open-field box (50 cm L × 50 cm W × 50 cm H) in a dark and soundproof environment. Mice were allowed to explore the apparatus freely for 10 min with a video camera recorded their movements. The total distance traveled were reported by an ANY-maze video tracking system (Stoelting, United Kingdom). The apparatus was thoroughly cleaned with 70% ethanol between the tests.

#### 2.4.2. Novel object recognition

**FIGURE 2:**
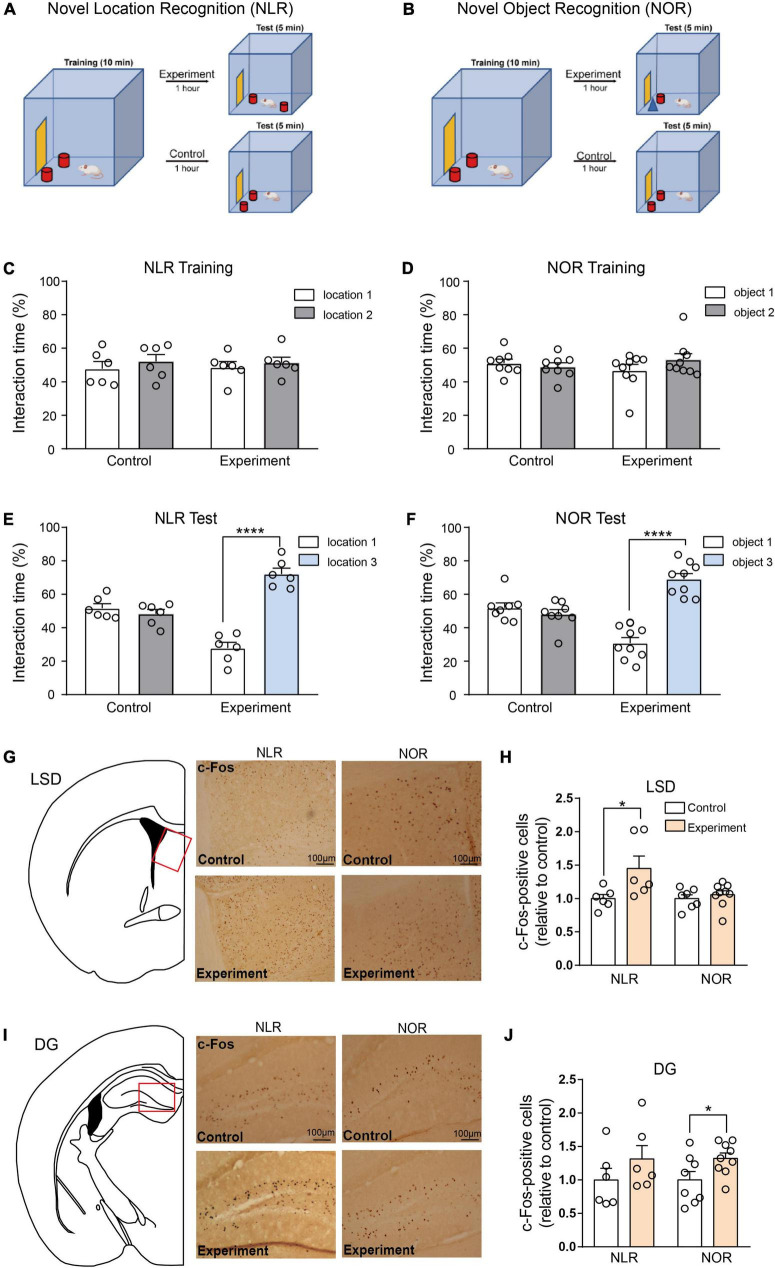
Lateral septal nucleus, dorsal part (LSD), and dentate gyrus (DG) involved in the regulation of novel location recognition (NLR) and novel object recognition (NOR), respectively. **(A,B)** The diagram of NLR and NOR tests. During the training phase, the mice explored two identical objects for 10 min. During the test phase, 1 h later, one of the objects was changed into a new object/location. The experimental mice were allowed to explore two distinct objects/locations for 5 min each. The control mice were still exposed to the same objects/locations as in the training phase. **(C)** The interaction time (%) of NLR during the training phase in control and experiment groups (*n* = 6 for each group). Unpaired *t*-test: control: *t*_(10)_ = 0.7176, *p* = 0.4894; experiment: *t*_(10)_ = 0.5592, *p* = 0.5884. **(D)** The interaction time (%) of NOR during the training phase in the control (*n* = 8) and experiment group (*n* = 9). Unpaired *t*-test: control: *t*_(14)_ = 0.6287, *p* = 0.5397; experiment: *t*_(16)_ = 1.269, *p* = 0.2227. **(E)** The interaction time (%) of NLR during the test phase in the control and experiment groups (*n* = 6 for each group). Unpaired *t*-test: control: *t*_(10)_ = 0.9233, *p* = 0.3776; experiment: *t*_(10)_ = 9.113, ****p < 0.0001. **(F)** The interaction time (%) of NOR during the test phase in control (*n* = 8) and experiment groups (*n* = 9). Unpaired *t*-test: control: *t*_(14)_ = 0.9937, *p* = 0.3373; experiment: *t*_(16)_ = 8.233, ****p < 0.0001. **(G)** Representative images of c-Fos positive cells in the LSD of control and experiment mice in NLR and NOR tests. Scale bar, 100 μm. **(H)** Quantification of c-Fos positive cells in the LSD of control and experiment mice in NLR and NOR tests (NLR: *n* = 6; NOR: *n* = 8). Unpaired *t*-test: NLR: *t*_(10)_ = 2.312, *p = 0.0434; NOR: *t*_(14)_ = 0.7066, *p* = 0.4914. All the data conformed to normality. **(I)** Representative images of c-Fos positive cells in the DG of control and experiment mice in NLR and NOR tests. Scale bar, 100 μm. **(J)** Quantification of c-Fos positive cells in the DG of control and experiment mice in NLR and NOR tests (NLR: *n* = 6; NOR: *n* = 8). Unpaired *t*-test: NLR: *t*_(10)_ = 1.177, *p* = 0.2663; NOR: *t*_(15)_ = 2.202, *p = 0.0437. All the data conformed to normality.

The NOR test was performed in a small open field box (25 cm L × 25 cm W × 25 cm H, one of whose walls was specially marked). Twenty-four hours before the training and test phases, the mice were habituated to the open field box for 10 min. During the training phase, the mice explored two identical objects defined as object 1 and object 2 for 10 min. This object was red wooden cylinder with a diameter of 3 cm and height of 6.5 cm (see Figure 2B). The exploration time for each object was manually calculated, specifically when the tip of the mouse’s nose was pointed at the target object and within a range of 2–3 cm from the target. The interaction time (%) was defined as object 1 or 2 investigation time / (object 1 investigation time + object 2 investigation time) × 100. During the test phase, 1 h later, object 2 was replaced with a new object defined as object 3. This object was a blue wooden cone with a diameter of 3 cm and a height of 6.5 cm (see Figure 2B). The experimental mice were allowed to explore object 1 and 3 for 5 min. The control mice were still exposed to object 1 and 2. The exploration time for each object is calculated. For the novel object, the interaction time (%) was defined as the object 3 investigation time / (object 1 investigation time + object 3 investigation time) × 100. And for the familiar object, the interaction time (%) was defined as the object 1 investigation time / (object 1 investigation time + object 3 investigation time).

#### 2.4.3. Novel location recognition

Similar to the NOR, the NLR test was also performed in the same small open field box. Mice were habituated to the open field box for 10 min 1 day before the training and test phases. During the training phase, the mice explored two identical objects positioned in location 1 (in the corner of the open field box, see Figure 2A) and location 2 (parallel to location 1, see Figure 2A) for 10 min. The exploration time for each object was calculated manually. The difference between the NOR and the NLR test occurred during the test phase, an hour after the training phase. The object in location 2 was moved to location 3 (on the diagonal of location 1, see Figure 2A). Control mice were explored at the same location as the training phase.

### 2.5. 3,3′-Diaminobenzidine staining and quantification

After the test phase of NLR or NOR, mice were deeply anesthetized by 0.5% pentobarbital sodium (100 mg/kg) and perfused intracardially with 4% paraformaldehyde in 0.1 M PBS. Specially, to stain the c-Fos, the above procedure should be carried out 60 min after the behavioral test. This 60-min interval is chosen as it falls within the typical period of peak production for c-Fos protein after a stimulus (da Silva et al., 2014). During this period, mice should be housed in a dark and quiet place. After removal, the brains were post-fixed with 4% (vol/vol) paraformaldehyde for 2–3 days at 4°C. Furthermore, they were dehydrated with 30% (wt/vol) sucrose, then sliced into coronal brain sections (30 μm) with a sliding microtome (Leica CM1950; Nussloch, Eisfeld, Germany). Subsequently, they were stored at −20°C in the cryoprotectant medium [30% glycerol, 30% ethylene-glycol in phosphate-buffered saline (PBS)] until sampling. The sections were rinsed with 1% PBS and PBS containing 0.5% Triton X-100 (PBS-Tx). Sections were incubated for 15 min with a solution containing 0.3% hydrogen peroxide and 10% methanol in PBS to quench endogenous peroxidase activity. The sections were then transferred to a blocking solution containing 10% normal goat serum, 1% milk, and 0.2% gelatin in PBS-Tx. After washing, sections were incubated with the primary antibody, rabbit anti-c-Fos (1:800, ab190289, Abcam), rabbit anti-NeuN (1:250, 26975-1-AP, Proteintech), and rabbit anti-doublecortin (1:500, 4604S, CST) at 4°C overnight. Subsequently, they were reacted with a secondary antibody [peroxidase-conjugated AffiniPure goat anti-rabbit Immunoglobulin G (H + L), 1:250, SA00001-2, Proteintech] for 2 h. Afterward, the sections were incubated with Streptavidin-Biotin Complex solution at 37°C for 30 min. Finally, they were incubated with ABC Vector Elite containing Avidin and Biotin in a dark environment.

To quantify the number of c- Fos-, NeuN-, or doublecortin (DCX)-positive cells, a series of systematically selected every 10th brain section was stained. The c-Fos-positive cells in the eight brain regions mentioned above (the hippocampus’s CA1, CA3, and DG, the LSD, the Cg1 and Cg2 of the ACC, Pir, and PRh) were counted by collecting five images spanning between bregma 1.10 and −3.16 mm under 10 × objective on a microscope. The NeuN-positive cells in the LSD, the NeuN- and DCX-positive cells in the DG were counted by collecting 5 images spaning between bregma 0.50 and −0.10 mm, bregma −1.46 and −3.16 mm, respectively, under 10 × objective on a microscope. The number of c-Fos-positive cells in eight different brain regions, the number of NeuN-positive cells in the LSD, and the number of DCX-positive cells in the DG were automatically counted within an area of 730 × 580 μm using the Find Maxima of ImageJ software. We measured the optical density of the NeuN-positive cells in the DG by ImageJ. The optical density value of NeuN-positive cells in the DG was quantified as the mean gray value of the slices. The outline of the brain regions mentioned above was drawn manually according to the mouse brain atlas (The Mouse Brain in Stereotaxic Coordinates, second edition). For statistical analysis, each mouse had 2–3 coronal sections per brain region. Furthermore, the average of individual measurements was used to calculate the group means. All immunostaining analyses were done blindly.

### 2.6. Statistical analysis

For all experiments, statistical analyses were performed using GraphPad Prism software. Data are presented as the mean ± SEM. The differences between the two means were assessed using unpaired *t*-tests. A two-way analysis of variance followed by Bonferroni’s *post-hoc* test, when appropriate, was used to analyze the NeuN-positive and DCX-positive cells with ibotenic acid and ASO treatment. Only values with *p* < 0.05 were accepted as statistically significant.

## 3. Results

### 3.1. Lateral septal nucleus, dorsal part, and dentate gyrus were related to the spatial and object recognition memory, respectively

To investigate the brain regions involved in regulating spatial and object recognition, two groups of mice were subjected to NLR and NOR tests, as shown in Figures 2A, B. During the training phase, no significant preference for one of the sides of the cage or for one of the two identical objects was observed in any of the mice (Figures 2C, D). During the test phase, the experimental group with two objects in different locations, spent more percentage of time interacting with the object in the novel location, whereas the control group with two objects in the same location as the training stage, mice showed no obvious preference (Figure 2E). Similar NOR test results were observed in our study (Figure 2F), suggesting spatial and object recognition memory in the experimental group.

Mice were sacrificed 1 h after the behavioral tests to determine which brain area participated in these behaviors. Furthermore, c-Fos staining was conducted in eight brain areas: the CA1, CA3, and DG of the hippocampus, the LSD, Cg1, and Cg2 of the ACC, the Pir and the PRh. An increase in the number of c-Fos-positive cells in the LSD test was found in the experimental group, while in the NOR test, there was no obvious difference in c-Fos-positive cells between the experimental and control groups (Figures 2G, H). In contrast, the number of c-Fos-positive cells was significantly increased in the DG in the experimental group compared with that in the control group in the NOR test. However, it did not increase in the NLR test (Figures 2I, J). Additionally, our results showed that the c-Fos-positive cells in the CA1 and CA3 of the hippocampus, the Cg1 and Cg2 of the ACC, the Pir and the PRh were not obviously different between the experimental and control groups in both the NLR and NOR tests (Supplementary Figure 1). These results, taken together, suggest that the LSD and DG (but not limited to) are necessary for regulating spatial and object recognition memory in the NLR and NOR tests, respectively.

### 3.2. Lesions of the LSD and DG induced by ibotenic acid contributed to deficits of spatial and object recognition memory, respectively

**FIGURE 3:**
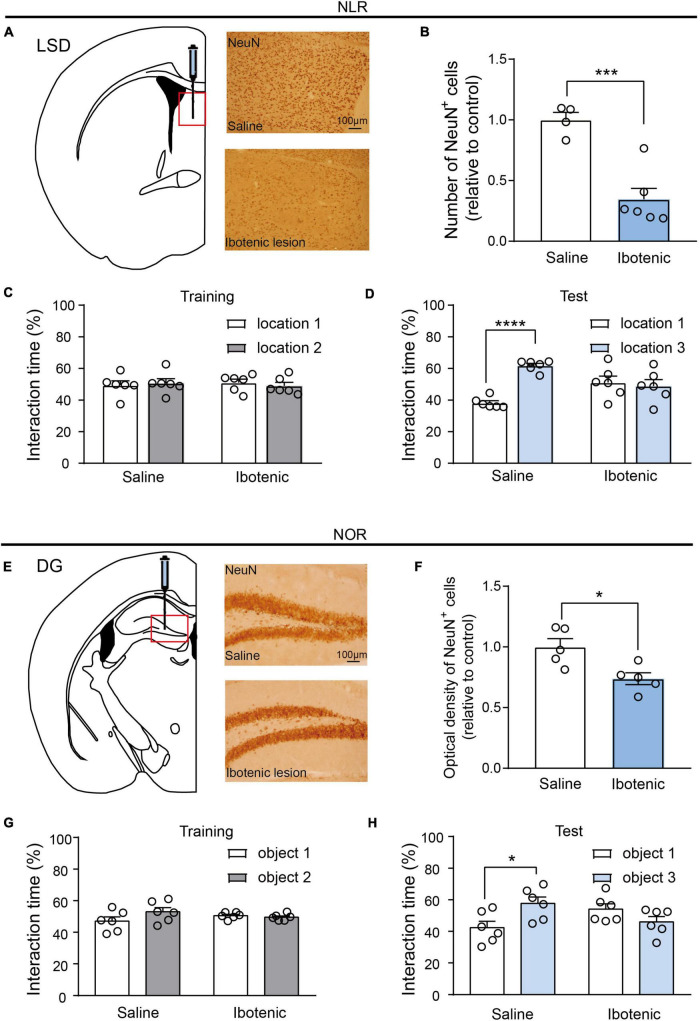
Lesions of LSD and DG induced by ibotenic acid contributed to deficits in spatial and object recognition memory. **(A)** Representative images of NeuN-positive cells in the LSD of saline and ibotenic lesioned mice. Scale bar, 100 μm. **(B)** The number of NeuN positive cells in the LSD of experiment mice. Ibotenic lesioned mice showed decreased neurons in LSD compared with saline mice (saline: *n* = 4; Ibotenic: *n* = 6). Unpaired *t*-test: *t*_(8)_ = 5.342, ***p = 0.0007. **(C)** The interaction time (%) of NLR during the training phase in saline (*n* = 6) and ibotenic lesioned mice (*n* = 6). Unpaired *t*-test: saline: *t*_(10)_ = 0.3271, *p* = 0.7504; Ibotenic: *t*_(10)_ = 0.6613, *p* = 0.5234. **(D)** The interaction time (%) of NLR during the test phase. Saline mice showed more preference for the novel location compared with the familiar location (*n* = 6). Unpaired *t*-test: *t*_(10)_ = 12.05, ****p < 0.0001. Ibotenic-lesioned mice showed no significant difference between the two locations. Unpaired *t*-test: *t*_(10)_ = 0.396, *p* = 0.7004. **(E)** Representative images of NeuN-positive cells in the DG of saline and ibotenic lesioned mice. Scale bar, 100 μm. **(F)** The optical density of NeuN positive cells in the DG of experiment mice. Ibotenic lesioned mice showed decreased neurons in DG compared with saline mice (saline: *n* = 5; Ibotenic: *n* = 5). Unpaired *t*-test: *t*_(8)_ = 3.111, *p = 0.0144. **(G)** The interaction time (%) of NOR during the training phase in saline (*n* = 6) and ibotenic lesioned mice (*n* = 6). Unpaired *t*-test: saline: *t*_(10)_ = 2.020, *p* = 0.0710; Ibotenic: *t*_(10)_ = 0.8544, *p* = 0.4129. **(H)** The interaction time (%) of NOR during the test phase. Saline mice showed more preference for the novel object compared with the familiar object (*n* = 6). Unpaired *t*-test: *t*_(10)_ = 2.863, *p = 0.023. Ibotenic-lesioned mice showed no significant difference between the two objects. Unpaired *t*-test: *t*_(10)_ = 1.680, *p* = 0.1239.

To further evaluate the importance of the LSD and DG brain regions, we performed bilateral injections of ibotenic acid into these regions. There were approximately 65 and 30% decreases in neuronal cells in the LSD and DG, respectively, (Figures 3A, B, E, F), suggesting successful lesions in the targeted brain regions. NLR and NOR tests were then conducted. Impaired spatial and object recognition memory was observed in the lesioned mice (Figures 3D, H), with no significant difference in the percentage of exploration time during the training phase (Figures 3C, G). This result was consistent with the conclusion drawn by a majority of experiments that object recognition memory to be impaired after temporary inactivation of the hippocampus (Ainge et al., 2006). It should be noted that object recognition memory would be unimpaired in rodents with permanent lesions of the hippocampus since the compensation of neural circuitry (Cohen and Stackman, 2015). Prior to the NLR and NOR tests, the OFT was conducted to confirm that the impaired recognition memory in lesioned mice was not an artifact of locomotor dysfunction. The results showed no difference in the total distance traveled by the saline and lesioned mice in the OFT (Supplementary Figure 2). Overall, these findings reinforce the critical role of LSD and DG for regulating spatial and object recognition memory.

### 3.3. ASO injection in the LSD region alleviated the spatial recognition memory impairment by functional neuronal regeneration

**FIGURE 4:**
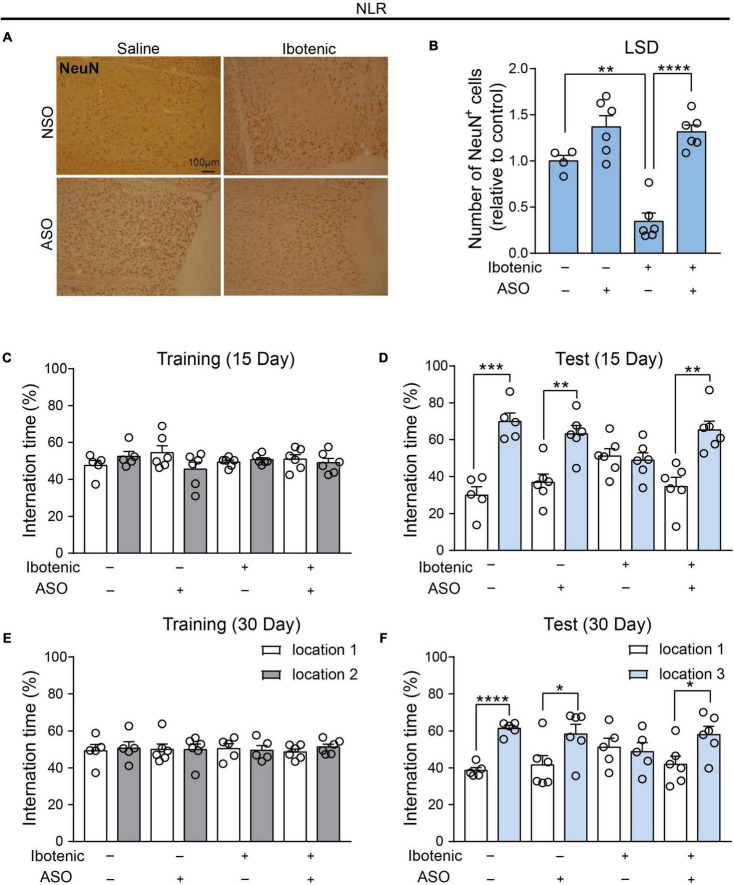
Antisense oligonucleotide injection rescued the spatial recognition memory in the lesioned mice by functional neuronal regeneration. **(A)** Representative images of NeuN-positive cells in the LSD. Scale bar, 100 μm. **(B)** The number of NeuN positive cells in the LSD (saline/NSO: *n* = 4; saline/ASO: *n* = 6; Ibotenic/NSO: *n* = 6; Ibotenic/ASO: *n* = 6). Two-way ANOVA: Ibotenic, *F*_(1,18)_ = 13.38, *p* = 0.0018; ASO, *F*_(1,18)_ = 47.77, *p* < 0.0001; interaction, *F*_(1,18)_ = 9.635, *p* = 0.0061; **p < 0.01, ****p < 0.0001 with Bonferroni’s *post-hoc* test. **(C)** The interaction time (%) of NLR during the training phase 15 days after ASO injection (saline/NSO: *n* = 5; saline/ASO: *n* = 6; Ibotenic/NSO: *n* = 6; Ibotenic/ASO: *n* = 6). Unpaired *t*-test: saline/NSO: *t*_(8)_ = 1.272, *p* = 0.2390; saline/ASO: *t*_(10)_ = 1.693, *p* = 0.1213; Ibotenic/NSO: *t*_(10)_ = 0.9252, *p* = 0.3766; Ibotenic/ASO: *t*_(10)_ = 0.5864, *p* = 0.5706. **(D)** The interaction time (%) of NLR during the test phase 15 days after ASO injection (saline/NSO: *n* = 5; saline/ASO: *n* = 6; Ibotenic/NSO: *n* = 6; Ibotenic/ASO: *n* = 6). Unpaired *t*-test: saline/NSO: *t*_(8)_ = 6.123, ***p = 0.0003; saline/ASO: *t*_(10)_ = 4.119, **p = 0.0021; Ibotenic/NSO: *t*_(10)_ = 0.3960, *p* = 0.7004; Ibotenic/ASO: *t*_(10)_ = 4.418, **p = 0.0013. **(E)** The interaction time (%) of NLR during the training phase 30 days after ASO injection (saline/NSO: *n* = 5; saline/ASO: *n* = 6; Ibotenic/NSO: *n* = 5; Ibotenic/ASO: *n* = 6). Unpaired *t*-test: saline/NSO: *t*_(8)_ = 0.3053, *p* = 0.7680; saline/ASO: *t*_(10)_ = 0.005915, *p* = 0.9954; Ibotenic/NSO: *t*_(8)_ = 0.2654, *p* = 0.7974; Ibotenic/ASO: *t*_(10)_ = 1.225, *p* = 0.2487. **(F)** The interaction time (%) of NLR during the test phase 30 days after ASO injection (saline/NSO: *n* = 5; saline/ASO: *n* = 6; Ibotenic/NSO: *n* = 5; Ibotenic/ASO: *n* = 6). Unpaired *t*-test: saline/NSO: *t*_(8)_ = 10.08, ****p < 0.0001; saline/ASO: *t*_(10)_ = 2.313, *p = 0.0433; Ibotenic/NSO: *t*_(8)_ = 0.3423, *p* = 0.7409; Ibotenic/ASO: *t*_(10)_ = 2.58, *p = 0.0274.

In our study, we investigated the potential therapeutic effect of ASO injection and NSO as control injection into the LSD of lesioned mice. An increased number of NeuN-positive cells in the LSD was found in the lesioned mice 30 days after ASO injection (Figures 4A, B), indicating neuronal recovery. During the training phase of NLR, no significant preference for one of the sides of the cage was observed in all mice (Figures 4C, E). Interestingly, we observed improved spatial recognition memory in lesioned mice after ASO injection for 15 days, as indicated by a larger percent of interaction time with the novel location object in the NLR test (Figure 4D). After another 15 days (30 days after injection of ASO), the spatial recognition memory of lesioned mice remained at normal levels compared with that of control mice (Figure 4F), suggesting that the therapeutic effect could be retained for some time. Importantly, the improvement in recognition memory was not due to the locomotor dysfunction, as there was no difference in the total distance traveled in the OFT among the groups (Supplementary Figure 3).

### 3.4. Adult-born neurons contributed to the improvement of object recognition memory in DG-lesioned mice

**FIGURE 5:**
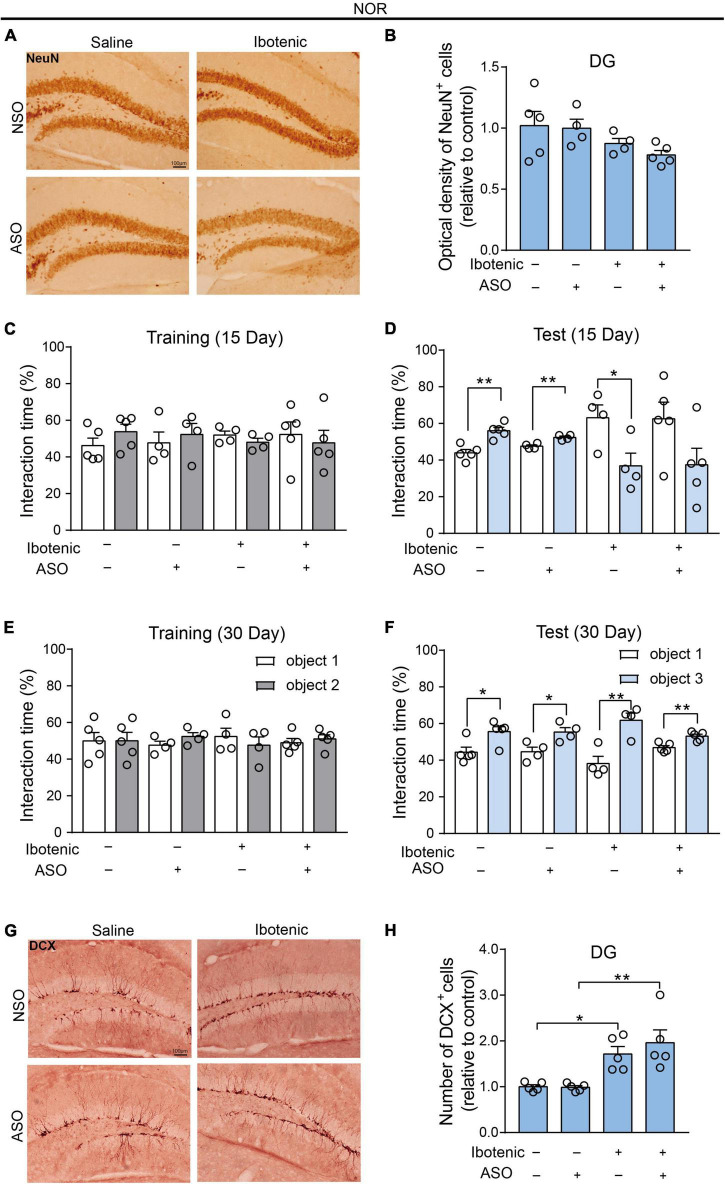
The adult-born neurons contributed to the improvement of object recognition memory in DG-lesioned mice. **(A)** Representative images of NeuN-positive cells in the DG. Scale bar, 100 μm. **(B)** The optical density of NeuN positive cells in the DG (saline/NSO: *n* = 5; saline/ASO: *n* = 4; Ibotenic/NSO: *n* = 4; Ibotenic/ASO: *n* = 5). Two-way ANOVA: Ibotenic, *F*_(1,14)_ = 5.367, *p* = 0.0362; ASO, *F*_(1,14)_ = 0.5271, *p* = 0.4798; interaction, *F*_(1,14)_ = 0.2095, *p* = 0.6542. **(C)** The interaction time (%) of NOR during the training phase 15 days after ASO injection (saline/NSO: *n* = 5; saline/ASO: *n* = 4; Ibotenic/NSO: *n* = 4; Ibotenic/ASO: *n* = 5). Unpaired *t*-test: saline/NSO: *t*_(8)_ = 1.305, *p* = 0.2282; saline/ASO: *t*_(6)_ = 0.5483, *p* = 0.6033; Ibotenic/NSO: *t*_(6)_ = 1.275, *p* = 0.2495; Ibotenic/ASO: *t*_(8)_ = 0.4711, *p* = 0.6502. **(D)** The interaction time (%) of NOR during the test phase 15 days after ASO injection (saline/NSO: *n* = 5; saline/ASO: *n* = 4; Ibotenic/NSO: *n* = 4; Ibotenic/ASO: *n* = 5). Unpaired *t*-test: saline/NSO: *t*_(8)_ = 4.744, **p = 0.0015; saline/ASO: *t*_(6)_ = 4.722, **p = 0.0033; Ibotenic/NSO: *t*_(6)_ = 2.672, *p = 0.0369; Ibotenic/ASO: *t*_(8)_ = 1.968, *p* = 0.0847. **(E)** The interaction time (%) of NOR during the training phase 30 days after ASO injection (saline/NSO: *n* = 5; saline/ASO: *n* = 4; Ibotenic/NSO: *n* = 4; Ibotenic/ASO: *n* = 5). Unpaired *t*-test: saline/NSO: *t*_(8)_ = 0.009471, *p* = 0.9927; saline/ASO: *t*_(6)_ = 1.555, *p* = 0.1710; Ibotenic/NSO: *t*_(6)_ = 0.7354, *p* = 0.4898; Ibotenic/ASO: *t*_(8)_ = 0.6312, *p* = 0.5455. **(F)** The interaction time (%) of NOR during the test phase 30 days after ASO injection (saline/NSO: *n* = 5; saline/ASO: *n* = 4; Ibotenic/NSO: *n* = 4; Ibotenic/ASO: *n* = 5). Unpaired *t*-test: saline/NSO: *t*_(8)_ = 2.923, *p = 0.0192; saline/ASO: *t*_(6)_ = 3.066, *p = 0.0220; Ibotenic/NSO: *t*_(6)_ = 4.267, **p = 0.0053; Ibotenic/ASO: *t*_(8)_ = 4.023, **p = 0.0038. **(G)** Representative images of doublecortin (DCX) positive cells in the DG. Scale bar, 100 μm. **(H)** The number of DCX-positive cells in the DG (saline/NSO: *n* = 5; saline/ASO: *n* = 5; Ibotenic/NSO: *n* = 5; Ibotenic/ASO: *n* = 5). Two-way ANOVA: Ibotenic, *F*_(1,16)_ = 26.67, *p* < 0.0001; ASO, *F*_(1,16)_ = 0.5129, *p* = 0.4842; interaction, *F*_(1,16)_ = 0.6266, *p* = 0.4402; *p < 0.05, **p < 0.01 with Bonferroni’s *post-hoc* test.

We injected ASO or NSO as controls into the DG brain region of lesioned mice. The NOR results in the training phase showed that there was no significant preference for two identical objects in all mice (Figures 5C, E). Moreover, the NOR results in the test phase demonstrated that the impaired object memory and improved object memory of the lesioned mice were observed 15 and 30 days after ASO injection, respectively (Figures 5D, F). This observation corresponded to the results showing no significant neuronal damage in the DG 30 days after injection (Figures 5A, B). Surprisingly, improved behavioral performance was also observed in the lesioned mice that received ibotenic acid into the DG but without ASO injection after 30 days (Figure 5F). Importantly, an increased number of DCX-positive cells, but not NeuN-positive cells, was found in DG-lesioned mice 30 days after ASO injection (Figures 5G, H), suggesting that adult-born neurons might contribute to the improvement of object recognition memory depending on the spontaneous regeneration of neurons in DG-lesioned mice. Additionally, there was no difference in the total distance traveled in the OFT among the groups (Supplementary Figure 3).

## 4. Discussion

Here, we focused on eight brain regions that are mostly associated with cognitive behavior, including the CA1, CA3, and DG of the hippocampus, the LSD, the Cg1 and Cg2 of the ACC, Pir and PRh. The results demonstrated that the LSD and DG were closely related to spatial and object recognition memory in the modified NLR and NOR tests. We further bilaterally lesioned these regions using excitotoxic ibotenic acid and found impaired spatial and object recognition memory. We then replenished damaged areas using the ASO strategy. By distinct mechanisms, the impaired spatial and object recognition memory of lesioned mice improved 40 days after ibotenic injection. Our results emphasized the crucial role of LSD and DG in regulating spatial and object recognition memory.

Consistent with the literature (Broadbent et al., 2004, 2010; Cohen and Stackman, 2015; Kesner et al., 2015; Bernstein et al., 2019), we found that in the present study, novelty and novel objects increase the number of c-Fos-positive cells in the DG, indicating that DG is crucial for the object recognition memory in the NOR test. It has been shown in rodents that adult hippocampal neurogenesis, which mainly occurs in the DG, is required for pattern separation and memory recognition (Sahay et al., 2011). Interestingly, improved behavioral performance in the NOR test was observed in the lesioned mice without ASO injection after 30 days (Figure 5F). In contrast, no enhanced performance was found in the NLR test of LSD-lesioned mice (Figure 4F). This observation corresponded to the results showing no significant neuronal damage in the DG after the injection of ibotenic acid for 40 days (Figures 5A, B).

Previous studies have reported that ASO can convert glial cells into functional neurons, including enhancing adult neurogenesis by consuming PTBP1. However, some studies have found that ASO-mediated astroglial PTBP1 repression could not achieve astrocyte-to-neuron conversion either in the hippocampus or striatum. Nevertheless, these studies speculate that some neurogenic cell types, such as neural stem cells, could be converted into neurons (Chen et al., 2022; Guo et al., 2022). Neural stem cells are located at the edge of the granular cell layer of the DG and produce neural progenitor cells (NPCs). These NPCs migrate along the subgranular area to produce granular neurons, thereby promoting hippocampal neurogenesis (Aimone et al., 2014). New and immature neurons are more flexible in establishing connections in the hippocampus than mature neurons, which is crucial for learning and memory (Encinas et al., 2013; Berg et al., 2019).

It is possible that adult-born neurons in the DG are responsible for this phenomenon that no effect on the NeuN-positive cell numbers in DG was observed in all groups. Adult-born neurons in the DG developed axons for 1–2 weeks following the final cell division and then fully integrate into the dentate circuitry one month after division (Overstreet et al., 2004). In our study, we observed impaired object recognition memory in mice 25 days after ibotenic injection. However, improved object recognition memory was observed in the lesioned mice, giving ibotenic acid to the DG after 40 days. Importantly, an increased number of DCX-positive cells was found in DG-lesioned mice 40 days after ibotenic injection (Figure 5H). These results prove that the restoration of object recognition memory is related to adult-born neurons in the DG.

In our study, there were no increase of c-Fos-positive cells in the CA1 and CA3 of hippocampus in the NLR or NOR test (Supplementary Figure 1). The findings are consistent with previous report that the c-Fos-positive cells were not significantly higher in the CA1 and CA3 brain regions after performing the object-based attention test (Wulaer et al., 2020). And we speculated that the NLR test might not be the best behavioral paradigm for detecting spatial memory associated with CA1 and CA3. Some studies and our recent results have shown that spatial memory in the Morris water maze or Barnes maze test is mostly related to the CA1 or CA3 regions (Zappa Villar et al., 2018; Bi et al., 2021; Xu et al., 2021). This observation indicated that the NLR test had functional limitations. More behavioral paradigms related to spatial memory should be assessed in future studies.

The lateral septal nucleus, which can be divided into dorsal, intermediate, and ventral parts (Swanson and Cowan, 1979), receives its major input from the hippocampal formation and regulates a variety of functions such as reward, feeding, anxiety, fear, sociability, and memory (Wirtshafter and Wilson, 2020; Rizzi-Wise and Wang, 2021). Specifically, the dorsal part of the lateral septal nucleus (LSD) is found to be involved in spatial recognition memory. This observation may be related to the projections received from the dorsal hippocampus, which regulates spatial learning and memory (Wirtshafter and Wilson, 2021). The septal nucleus also projects to the hippocampus, mediating the exploration, spatial learning, and locomotion (An et al., 2021). In our study, we found impaired behavioral performance in the NLR test after lesioning the LSD using excitotoxic ibotenic acid (Figure 3D). After regenerating neurons in the LSD, improved spatial recognition memory was observed (Figure 4F). These data indicate that LSD is important and necessary for the regulation of spatial recognition.

The ASO strategy was used in the regeneration of neurons in LSD- and DG-lesioned mice by distinct mechanisms. However, the Ibotenic/ASO group exhibited less neuronal regeneration than the Ibotenic/NSO group. Further studies are needed to fully understand the mechanisms underlying the restoration of object recognition memory in DG-lesioned mice.

## 5. Conclusion

We have demonstrated that LSD and DG are involved in spatial and object recognition through modified NLR and NOR tests. Notably, these two brain regions have close associations with the hippocampus, which further highlights the hippocampus’s crucial role in memory processing. Thus, our findings provide a more comprehensive understanding of the specific brain regions involved in different forms of recognition memory and offer potential targets for therapeutic interventions to improve impaired spatial and object recognition memory.

## Data availability statement

The original contributions presented in this study are included in the article/Supplementary material, further inquiries can be directed to the corresponding authors.

## Ethics statement

The animal study was reviewed and approved by the Animal Care and Use Committees of Ningbo University, China (Reference Number: 10099).

## Author contributions

XZha and YJ designed the study and wrote the protocol. XZha, YJ, and FD wrote the first draft of the manuscript. FD, YJ, YD, XZ, LP, LH, LeX, and XX conducted the experiments. All authors contributed to the article and approved the submitted version.
