# Supplementary material for: Lateral septal nucleus, dorsal part, and dentate gyrus are necessary for spatial and object recognition memory, respectively, in mice
Source: Front Behav Neurosci. 2023 Mar 31;17:1139737. doi: 10.3389/fnbeh.2023.1139737 (PMC10102498; doi:10.3389/fnbeh.2023.1139737)
Supplement: Supplementary file 1 [file Data_Sheet_1.docx]

Supplementary Material

Lateral septal nucleus, dorsal part, and dentate gyrus are necessary for spatial and object recognition memory, respectively, in mice

Ying-Ke Jiang ^1†^, Fei-Yuan Dong ^1†^, Yi-Bei Dong ^1^, Xin-Yi Zhu ^1^, Lu-Hui Pan ^1^, Lin-Bo Hu ^1^, Le Xu ^1^, Xiao-Fan Xu ^1^, Li-Min Xu ^2^*, Xiao-Qin Zhang ^1^*

*** Correspondence:**

Dr. X.Q. Zhang and Li-Min Xu,

Department of Pharmacology

Medical School of Ningbo University, 818 Fenghua Rd, Ningbo, Zhejiang 315211, China

Tel: +86-574-87609580; Fax +86-574-87608638

Email: [zhangxiaoqin1@nbu.edu.cn](mailto:zhangxiaoqin1@nbu.edu.cn) or [xuliminbaby@163.com](mailto:xuliminbaby@163.com)

##
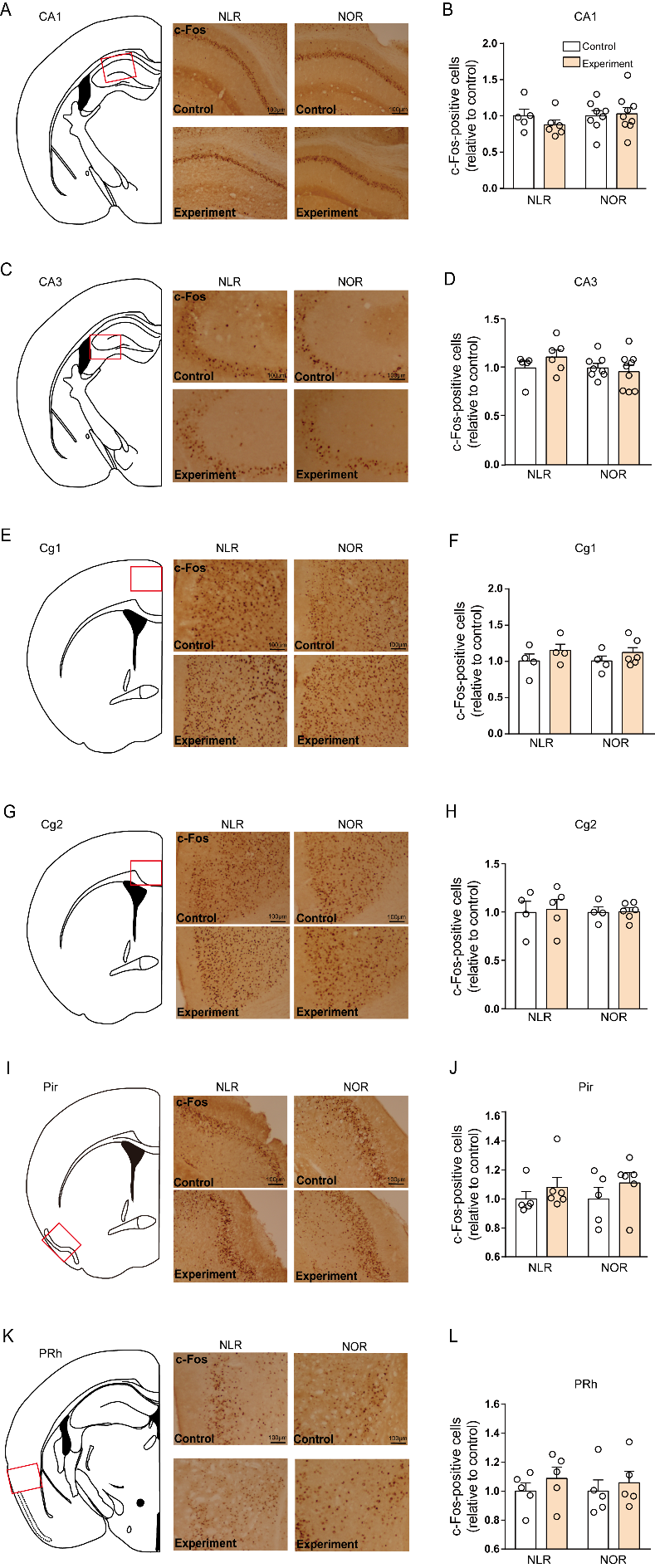
Supplementary Figures

**Figure S1. Expression of c-Fos in 5 different brain regions in mice of novel location recognition (NLR) or novel object recognition (NOR) tests. Related to Figure 3.**

**(A)** Typical images of c-Fos-positive cells in the CA1 of control and experimental mice in the NLR and NOR tests. Scale bar, 100 μm.

**(B)** The number of c-Fos-positive cells in the CA1 of control and experimental mice in the NLR (control: n = 5; experiment: n =6) and NOR (control: n = 8; experiment: n = 9). Unpaired t test: t _(9)_ = 1.108, p = 0.2964; t _(15)_ = 0.2574, p = 0.8004.

**(C)** Typical images of c-Fos-positive cells in the CA3 of control and experimental mice in the NLR and NOR tests. Scale bar, 100 μm.

**(D)** The number of c-Fos-positive cells in the CA3 of control and experimental mice in the NLR (control: n = 5; experiment: n =6) and NOR (control: n = 8; experiment: n = 9). Unpaired t test: t _(9)_ = 1.191, p = 0.2641; t _(15)_ = 0.5077, p = 0.6191.

**(E)** Typical images of c-Fos-positive cells in the Cg1 of control and experimental mice in the NLR and NOR tests. Scale bar, 100 μm.

**(F)** The number of c-Fos-positive cells in the Cg1 of control and experimental mice in the NLR (control: n = 4; experiment: n =4) and NOR (control: n = 4; experiment: n = 6). Unpaired t test: t _(6)_ = 1.069, p = 0.3264; t _(8)_ = 1.08, p = 0.3117.

**(G)** Typical images of c-Fos-positive cells in the Cg2 of control and experimental mice in the NLR and NOR tests. Scale bar, 100 μm.

**(H)** The number of c-Fos-positive cells in the Cg2 of control and experimental mice in the NLR (control: n = 4; experiment: n =5) and NOR (control: n = 4; experiment: n = 6). Unpaired t test: t _(7)_ = 0.2165, p = 0.8348; t _(8)_ = 0.1253, p = 0.9034.

**(I)** Typical images of c-Fos-positive cells in the piriform nucleus (Pir) of control and experimental mice in the NLR and NOR tests. Scale bar, 100 μm.

**(J)** The number of c-Fos-positive cells in the Pir of control and experimental mice in the NLR (control: n = 5; experiment: n =6) and NOR (control: n = 5; experiment: n = 6). Unpaired t test: t _(9)_ = 0.9028, p = 0.3901; t _(9)_ = 1.053, p = 0.3199.

**(K)** Typical images of c-Fos-positive cells in the perirhinal cortex (PRh) of control and experimental mice in the NLR and NOR tests. Scale bar, 100 μm.

**(L)** The number of c-Fos-positive cells in the PRh of control and experimental mice in the NLR (control: n = 5; experiment: n =5) and NOR (control: n = 5; experiment: n = 5). Unpaired t test: t _(8)_ = 0.9213, p = 0.3838; t _(8)_ = 0.5244, p = 0.6142.


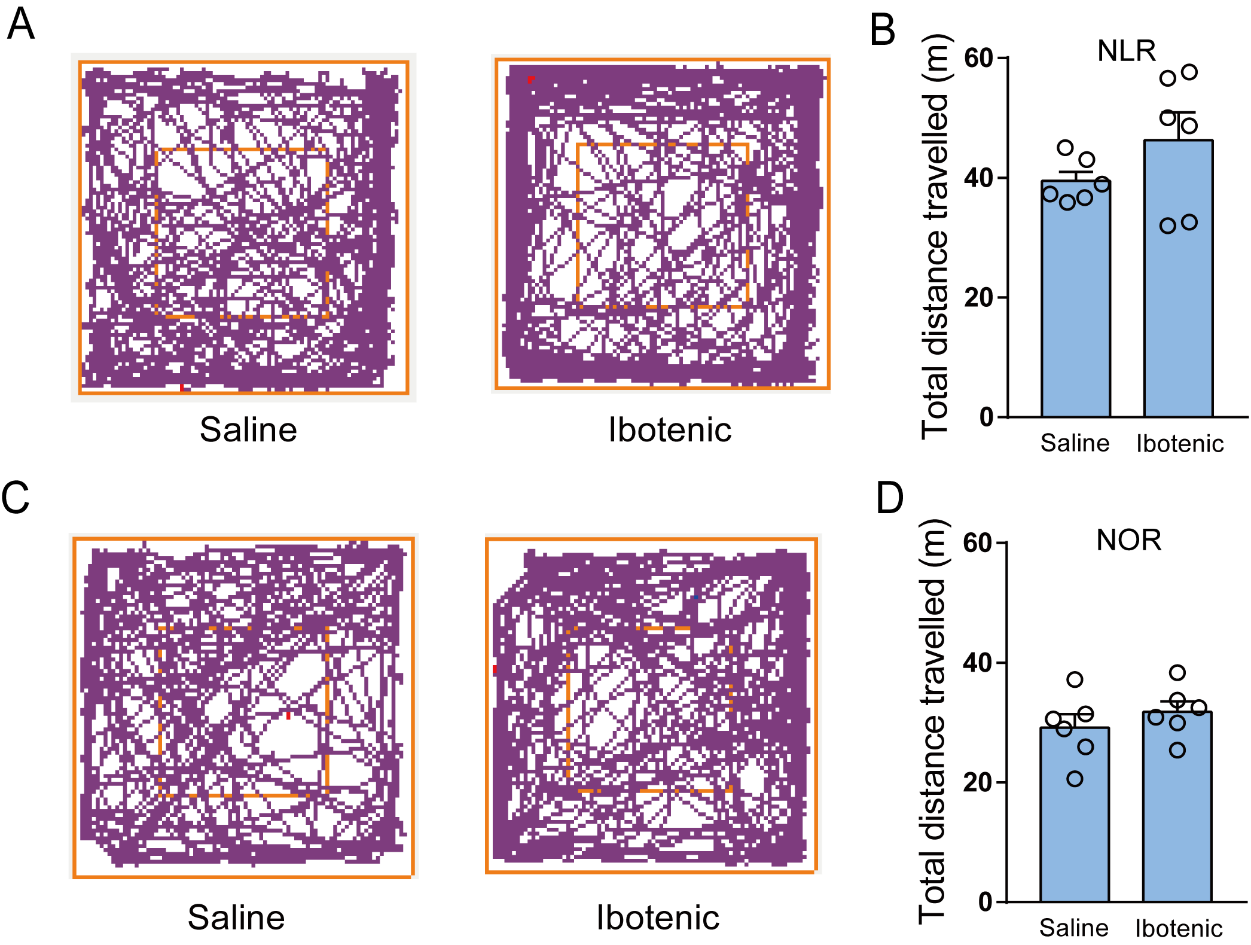


**Figure S2. Normal locomotor function in the lesioned mice. Related to Figure 4.**

**(A-B)** The total distance traveled by saline (n = 6) and ibotenic (n = 6) mice in the NLR test. Unpaired t-test: t _(10)_ = 1.389, p = 0.1950.

**(C-D)** Total distance traveled by saline (n = 6) and ibotenic (n = 6) mice in the NOR test. Unpaired t-test: t _(10)_ = 0.9328, p = 0.3729.


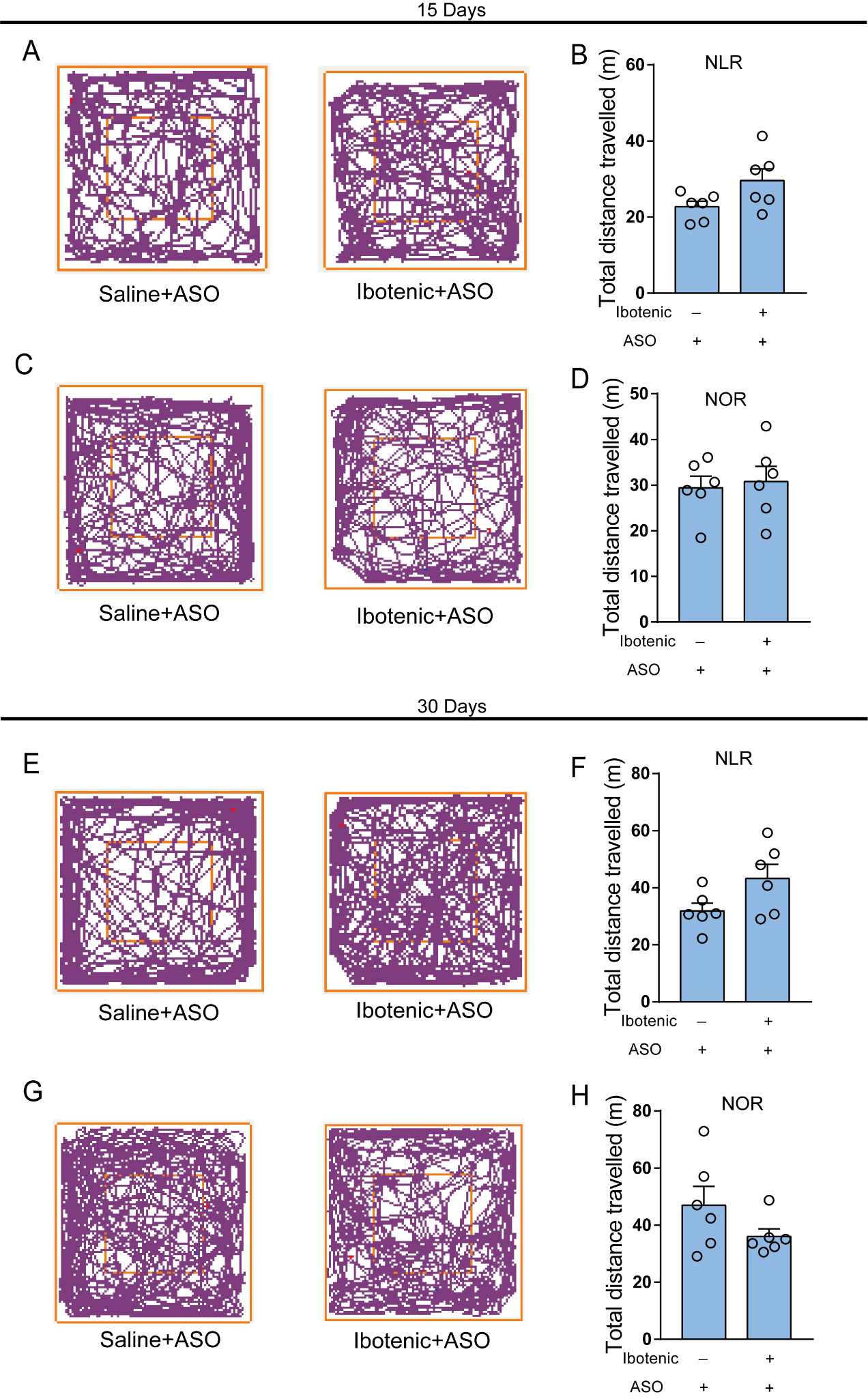


**Figure S3. Normal locomotor function in mice with the injection of Ibotenic or antisense oligonucleotide (ASO). Related to Figure 5-6.**

**(A-B)** Total distances traveled by saline/ASO (n = 6) and ibotenic/ASO (n = 6) mice 15 d after ASO injection in the NLR test. Unpaired t-test: t _(10)_ = 2.032, p = 0.0696.

**(C-D)** Total distances traveled by saline/ASO (n = 6) and ibotenic/ASO (n = 6) mice 15 days after ASO injection in the NOR test. Unpaired t-test: t _(10)_ = 0.3219, p = 0.7541.

**(E-F)** Total distances traveled by saline/ASO (n = 6) and ibotenic/ASO (n = 6) mice 30 days after ASO injection in the NLR test. Unpaired t-test: t _(10)_ = 2.037, p = 0.0690.

**(G-H)** Total distances traveled by saline/ASO (n = 6) and ibotenic/ASO (n = 6) mice 30 d after ASO injection in the NOR test. Unpaired t-test: t _(10)_ = 1.555, p = 0.1510.
